# Comparison of Three Groups of Patients Having Low Dose Rate Prostate Brachytherapy: Prostate-Specific Antigen Failure and Overall Survival

**DOI:** 10.7759/cureus.18185

**Published:** 2021-09-22

**Authors:** Jacquelyn Booher, Peter Domenig, Benjamin Goldman, Todd Campbell, Kyle Verdecchia, Judith A Boura, Paul J Chuba

**Affiliations:** 1 Urology, Ascension Macomb-Oakland Hospital, Warren, USA; 2 Urology, Ascension Macomb Oakland Hospital, Warren, USA; 3 Radiation Oncology, Henry Ford Hospital Detroit, Detroit, USA; 4 Research, Ascension Macomb-Oakland Hospital, Warren, USA; 5 Radiation Oncology, Ascension Macomb-Oakland Hospital, Warren, USA

**Keywords:** low dose rate prostate brachytherapy, prostate cancer, radiation therapy, radiation physics, seed implant

## Abstract

Purpose

To examine dosimetric and clinical outcomes for Cs-131 radioactive seed implant compared to Pd-103 and I-125.

Background/Significance

Cs-131 is a novel isotope with relatively short half-life (9.7 days) that may have clinical advantages in seed implant treatments of prostate cancers. There may be a shorter duration of symptoms and increased PSA control rates.

Methods

We performed a retrospective study in which clinical and dosimetric outcomes were compared for 186 prostate implants performed over a ten-year time period at three different Ascension hospitals. Isotopes that were used included Cs-131 (n=66; half-life 9.7 days), I-125 (n=60; half-life 60 days), and Pd-103 (n=60; half-life 17 days)

Results

The implants used standard radiation dosages. These were 145 Gy for I-125 alone or 109 Gy when combined with external beam radiation. In the case of Cs-131 used alone, the dose was 115 Gy or 85 Gy when combined with an external beam. For Pd-103, 125 Gy was used for monotherapy and 90 Gy when combined with an external beam. The Cs-131 dosimetry was found to be similar to I-125 and Pd-103 on a quantitative basis. However, there was better homogeneity, and the delivered activity per seed and the number of seeds employed were greater compared to other isotopes. We compared the corrected total source strengths (i.e. normalized to sample mean values) and were able to demonstrate similar distributions for the three isotopes. Dosimetric analysis also suggested there was superior homogeneity with Cs-131. The median PSA value at 60 months was 0.11 ng/ml. There were only a few PSA failures in the three groups of cases, nonetheless, the Cs-131 had the fewest.

Conclusions

One attractive option for men with early-stage prostate cancer is interstitial brachytherapy. The use of the shorter-acting Cs-131 isotope may be expected to have dose-related side effects that resolve more rapidly. This series suggests a trend for improved PSA control outcomes for Cs-131 patients compared with I-125 and Pd-103.

## Introduction

Prostate cancer is the second most common cause of cancer in men and can be associated with significant morbidity and mortality [1]. One treatment pathway for localized prostate cancer has been permanent prostate seed implant or low dose rate (LDR) brachytherapy [2,3]. This minimally invasive approach has been associated with long-term disease control outcomes that appear to be equivalent to prostatectomy and external beam radiation therapy (EBRT) for low-risk and select intermediate-risk patients [3]. In higher-risk disease, the combination of hormone therapy, external beam radiation, and LDR brachytherapy is associated with superior failure-free and overall survival compared with the combination of hormone therapy and external beam radiation alone [4,5]. 

Historically, the two most commonly used isotopes for LDR brachytherapy have been Iodine-125 (I-125) and Palladium-103 (Pd-103) which have a half-life of 61 and 17 days, and energy of 28 KeV and 21 KeV respectively [5]. Cesium-131 is a novel radioisotope with a shorter half-life of 9.7 days and higher energy (30.4KeV) which has been suggested to confer possible clinical advantages of increased disease control and shorter duration of symptoms [6-10]. In this report, we examine and compare the dosimetry, PSA failure rates, and overall survival for patients treated with Cs-131, in comparison with I-125, and Pd-103 brachytherapy in a community hospital system.

## Materials and methods

The data collection interval was from 2007 to 2017 yielding 66 patients with localized prostate cancer who received LDR prostate implant using Cs-131 at Ascension Providence Rochester Hospital, Ascension Macomb-Oakland Hospital, and Ascension St. John Hospital. A comparison cohort consisting of a group of patients treated using I-125 (n=59), and Pd-103 (n=61) LDR implants having a minimum PSA follow-up of 24 months were selected for evaluation of dosimetry and clinical outcomes. Data was collected as part of an IRB-approved retrospective study. 

The radiation doses used for implant were as follows: for I-125 used alone (monotherapy), the dose was 145 Gy, and when combined with external radiation the dose was 109 Gy. The dose for Cs-131 was 115 Gy when used alone and 85 Gy when combined with external radiation therapy (1.8 to 2.0 U activity). In the case of Pd-103, the dose when used alone was 125 Gy, or 90 Gy when combined with external radiation. Post-implant dosimetric information was also collected with parameters reviewed including prostate volume, number of seeds, delivered activity, and total activity. The percent dose received by 90 percent of the prostate (D90), and the percent volume of the prostate receiving 100, 150, and 200 percent of the prescribed dose (V100, V150, and V200) was recorded. The addition of external beam radiation included 45 Gy given in 1.8 Gy daily fractions using 3-dimensional conformal or intensity-modulated radiation therapy methods. For combination therapy, the prostate, seminal vesicles, and (in some patients) the pelvic lymph nodes were treated. 

PSA failure and overall survival were calculated for each isotope treatment group. The patients were risk-stratified according to the National Comprehensive Cancer Network (NCCN) definitions. Overall survival was based in part on death certificate data obtained from the State of Michigan through the Michigan Cancer Foundation and the Karmanos Cancer Institute. Kaplan-Meier estimates of PSA failure were generated using the ‘Phoenix’ definition of nadir PSA plus 2 for biochemical failure [11]. Case characteristics were collected in order to determine the D’Amico risk category for each patient. All analyses were completed using SAS for Windows® 9.4, Cary, NC. Kaplan-Meier plots were also completed for each of the 3 different isotope groups for overall survival. The categorical variables were examined with Chi-square tests where appropriate (expected frequency >5 in 80% of cells); otherwise, Fisher’s Exact tests were used. Age was compared using the Student's t-test. The remaining continuous variables were analyzed with Wilcoxon rank sum tests since none appeared to be normally distributed for the groups.

## Results

The study included 186 patients who underwent prostate brachytherapy. There were 66 patients with Cs-131 implantation, 59 with I-125, and 61 with Pd-103. The mean pretreatment PSA values for the three groups were Cs-131 5.73 ng/ml, I-125 6.62 ng/ml, and for Pd-130 8.87 ng/ml. The three implant groups were selected to have adequate follow-up for analysis (median follow-up was 37 months for PSA failure-free survival and 40 months for overall survival). but were relatively well-matched (but not matched in advance) with respect to age, grade, and risk category (Table 1). 

**Table 1:** Patient and Tumor Characteristics *chi-squared

		Cs-131 (n=66)	I-125 (n=59)	Pd (n=61)	p-value
Age	Mean (SD)	68 (8.0)	69 (7.8)	70 (6.0)	0.35
	Median age	69	71	70	
	Minimum to Maximum	48 to 79	48 to 81	59 to 82	
Race n (%)	White	52 (80.0)	49 (83.1)	42 (84)	0.29
	Black	13 (20.0)	9 (15.3)	6 (12)	
	Other	0 (0.0)	1 (1.7)	2 (4)	
Max Gleason Sum n (%)	6	31 (47.7)	37 (62.7)	17 (27.9)	0.0007*
	7	33 (50.8)	19 (32.2)	39 (63.9)	
	8	1 (1.2)	2 (3.4)	5 (8.2)	
	9	0 (0.0)	1 (1.7)	0 (0.0)	
Stage	T1c	50 (78.1)	48 (81.4)	17 (27.9)	<0.0001*
	T2a	11 (17.2)	9 (15.3)	21 (34.4)	
	T2b	0.0 (0.0	1 (1.7)	13 (21.3)	
	T2c	3 (4.7)	1 (1.7)	8 (13.1)	
	T3	0 (0.0)	0 (0.0)	2 (3.3)	
Pretreatment PSA	0-10	51 (98.1)	52 (88.1)	48 (80)	0.03*
	10-20	1 (1.9)	6 (10.2)	9 (15)	
	>20	0	0	3 (5)	
D’Amico Risk Category	Low	27	33	14	0.04*
	Intermediate	26	18	25	
	High	1	4	4	
Combination EBRT		25	12	17	0.03*
Adjuvant Hormone Therapy		3	11	20	<0.0001*
Number of Seeds	Median	64	74	66	0.008*
	Minimum to Maximum	38 to 98	41 to 109	32 to 129	
Prostate Volume (cc)	Median	33	29	29	0.059
	Minimum to Maximum	15 to 61	15 to 58	14 to 61	

Summary of the pretreatment characteristics for the three groups are shown in Table 1. The median age at diagnosis for the Cs-131 group was 69 years, similar to the I-125 (71 years) and Pd-103 (70 years). There were significant differences among the three groups with respect to Gleason grade (p=0.007), clinical stage (p<0.0001), and pretreatment PSA (p=0.03) with the highest risk group being Pd-103. Very many patients treated with I-125 and Cs-131 had low risk by the D’Amico category. For patients who underwent Cs-131 prostate brachytherapy, 31 had Gleason Grade 6 (47.7%), 33 had Gleason Grade 7 (50.8%), and one patient had Gleason Grade 8 (1.2%) cancer. Of these patients, 61 had stage T1c or T2a (95.3%) and 3 had T2c (4.7%) stage cancer.

For the I-125 patient group, there were 37 patients with Gleason Grade 6 (62.7%), 19 with Gleason Grade 7 (32.2%), and 3 patients with Gleason Grade 8 or 9 (5.1%) cancer. Fifty-seven cases (96.7%) had stage T1c or T2a, and 2 cases (3.4%) has stage T2c cancer. For the Pd-103 group, there were 17 patients with Gleason Grade 6 (27.9 %), 39 with Gleason Grade 7 (63.9%), and 5 with Gleason Grade 8 or 9 (8.2%) cancer. There were 38 (62.3%) stage T1c/T2a, 21 (34.4%) T2b/T2c, and 2 cases (3.3%) stage T3 cancer. Combination therapy was utilized in 25 out of the 66 Cs-131 patients with 4500 cGy EBRT. There were 12 of the Pd-103 patients and 17 of the I-125 patients who had combination of 4500 cGy EBRT with brachytherapy. 

**Figure 1:**
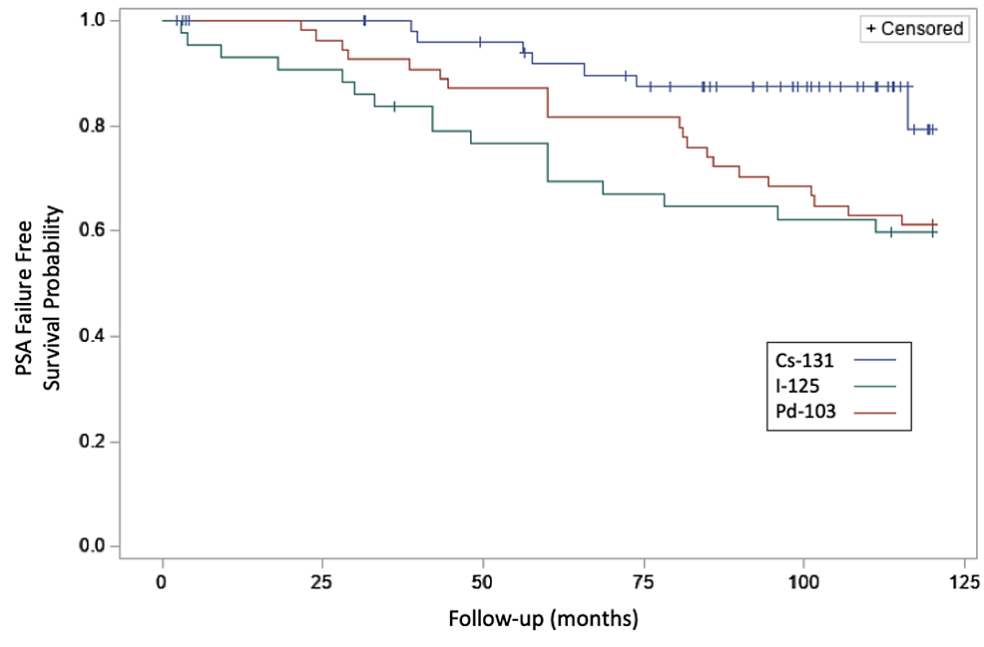
PSA Failure Free Survival for Three Groups of Implant Patients Kaplan Meier curves of PSA failure free survival are shown for three groups of prostate seed implant patients.  The upper curve (blue) shows the result for Cs-131 cases, the middle curve (red) shows the result for Pd-103 cases, and the lower curve (green) shows the result for  I-125 cases.

The median follow-up was 37 months for PSA failure-free survival and 40 months for overall survival. PSA failure rates were compared between the three isotope groups. There were very few PSA failures overall, and zero were noted in the Cs-131 group compared to 11 (9.2%) PSA failures combined between the I-125 and the Pd-103 groups. The median PSA value at 60 months was 0.11 ng/ml for all groups. PSA failures were greater with increasing Gleason grade (data not shown). In Figure 1, the PSA failure free survival curves for each of the three groups are shown. The Cs-131 group had improved PSA failure free survival which did appear to reach statistical significance in comparison to each of the other isotopes (p=0.04) but this could not be adjusted by risk category (data not shown). The freedom from PSA failure rate at 36 months was 100%, 95.6%, and 95.2% for the Cs-131, I-125, and Pd-103 groups respectively.

**Figure 2:**
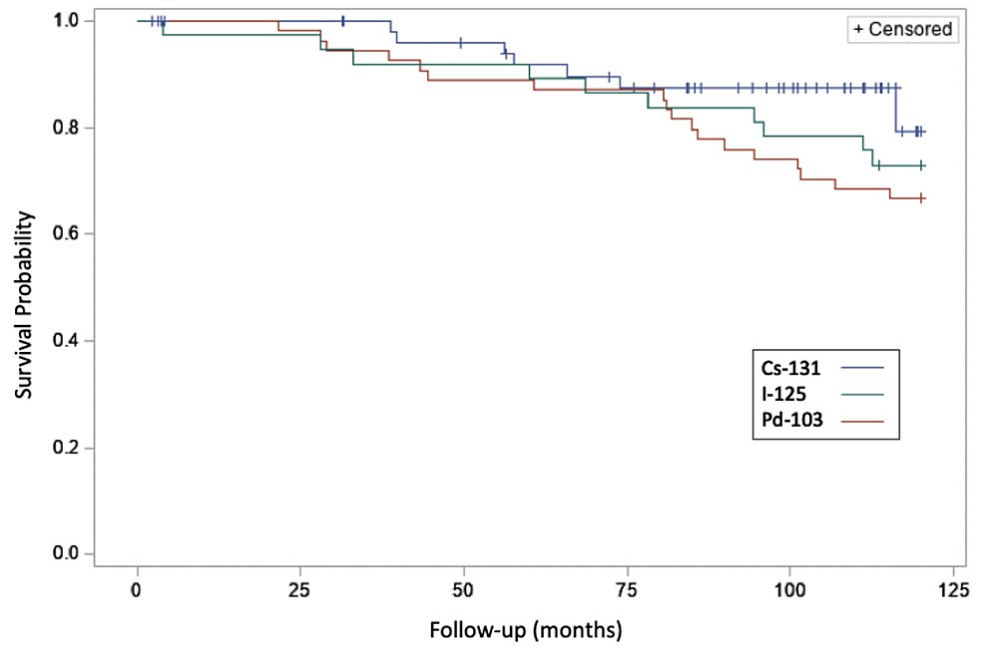
Overall Survival for Three Groups of Implant Patients Kaplan Meier curves illustrating the overall survival for three groups of patients having seed implants are shown.  The upper curve (blue) shows the result for Cs-131 cases, the middle curve (green) shows the result for I-125 cases, and the lower curve (red) shows the result for Pd-103 cases.

The Kaplan Meier curves of overall survival for the three groups are shown in Figure 2. At the conclusion of the data collection interval, a total of 65 patients were deceased, and 87 patients were living. For the patients who received Cs-131, 8/66 (12%) were deceased. Considering the I-125 and Pd-103 groups, there were 57/120 (48%) deceased. The 5-year overall survival was 91.7%, 88.9%, 89.2% for the Cs-131, I-125, and Pd-103 groups respectively. Although the overall survival of the Cs-131 group was higher compared to the other two groups, statistical significance was not reached (p=0.25). 

**Figure 3:**
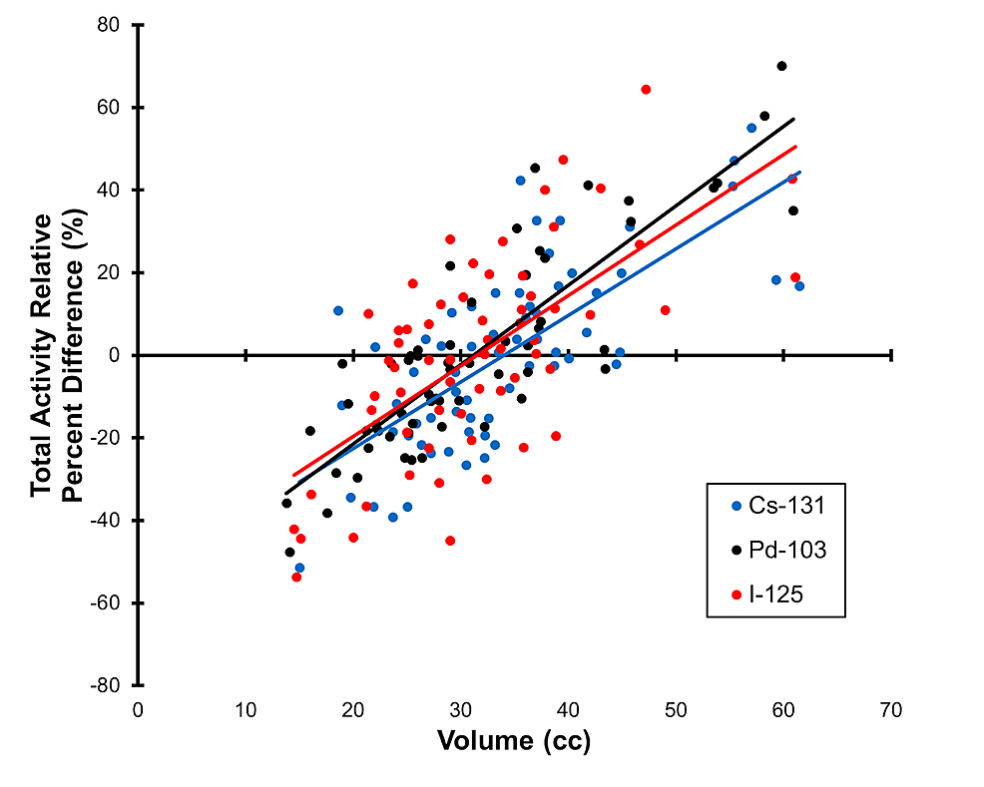
Dosimetric Analysis of Prostate Seed Implants with Three Different Isotopes Dosimetry for individual seed implants is illustrated by plotting total activity versus implant volume.  The three groups compared are Cs-131 (blue), Pd-103 (black) and I-125 (red).

With respect to the dosimetric analysis, Cs-131 dosimetry showed better homogeneity (Figure 3). There was greater activity delivered per seed and a greater number of seeds used compared to other isotopes. As shown here, the total source strength (which was normalized to sample mean values) showed a similar distribution for the three isotopes (Figure 3). A comparison of the V150 (mean percent) showed 36% for I-125 and 27% for Cs-131 (p<0.0001) which suggests that the homogeneity for Cs-131 is better. The percent V200 was 13% vs 12 % (p=0.012) for I-125 versus Cs-131. The D90 for Cs-131 versus I-125 was 98% versus 92%, respectively (p<0.001). As expected, the total source strength was related to prostate volume for all the isotopes showing similar treatment planning (data not shown).

## Discussion

The Cs-131 implant for prostate cancer was FDA approved in 2003 and to date, there are just a few reports describing long-term outcomes. As the utilization of this treatment option increases, it is important that safety and efficacy, as well as dosimetric equivalence, is studied in comparison to standard therapy. This will facilitate Cs-131 brachytherapy as an option available to a broader patient population outside of large and academic institutions. 

The proposed clinical advantages for the use of Cs-131 are related to its energy and half-life [8-10]. In particular, the shorter half-life might be expected to limit the duration of acute urinary morbidity and may be more effective at targeting mitotically active cancer cells. This is because an isotope with shorter half-life may theoretically be more effective in killing higher grade tumors having shorter potential doubling time [7]. In practice, it is unclear whether this effect is clinically relevant. 

In this study, we report our experience introducing Cs-131 low dose rate brachytherapy for prostate cancer and compare with patients treated with I-125 and Pd-103. Given the retrospective nature of this study, the three groups were not matched in advance and the risk category for Cs-131 and I-125 cases was lower (Table 1). This is likely related to the fact that during the time period in which Pd-103 was used, generally higher risk cases were selected (Table 1). Fortunately, we found few PSA failures across all three isotope groups. It is notable the data suggests a lower rate of PSA failure for Cs-131 and a statistically significant difference compared to the Pd-103 and I-125 groups. However, this may be attributed to differences in risk categories between groups. 

Clinical advantages for the Cs-131 isotope may increase the popularity of low dose rate brachytherapy for non-metastatic prostate cancers. The Ascende-RT trial [4] provided randomized data showing improved cancer control outcomes for intermediate and high-risk patients with the addition of low dose-rate brachytherapy to the combination of hormone therapy and external beam radiation. At a median follow-up of 6.5 years, it was noted that within the brachytherapy arm of the trial, there was increased rates of grade 3 GU events with 5 year cumulative incidence of 8.6% vs 2.2% (p=0.058) and slightly greater grade 3 GI morbidity (8.1% vs 3.2%; p=0.124).

Tomaszewski et al. [12] first compared initial PSA outcomes in men undergoing Cs-131 prostate brachytherapy to men treated with I-125. They found that both isotopes produced equivalent reduction in PSA levels at 3 and 6 months following treatment. The mean PSA for both groups was 7.3 pretreatment and initial post-treatment PSA was 1.5 in the I-125 group and 1.2 in the Cs-131 group. 

Two recent large single arm studies have now confirmed the use of Cs-131 as an effective alternative isotope. Moran et al. [13] reported on 571 cases of Cs-131 prostate brachytherapy performed at the Prostate Cancer Foundation of Chicago. The five-year freedom from biochemical failure rates were 96.9% for low-risk, 92.8% for intermediate-risk, and 93.2% for high-risk patients, respectively. Similarly, Benoit et al. [14] reported on analysis of 669 men who had Cs-131 prostate implant at the University of Pittsburgh. Using the Phoenix criteria, biochemical freedom of disease was 97.1 % at 5 years for low-risk, 94% for intermediate-risk, and 86.2% for high-risk cases. At 10 years, the rates were 95.3%, 90.1%, and 56.6%. In both groups, large numbers of intermediate and high-risk patients had combination treatment with brachytherapy and external beam radiotherapy. Although the current study is much smaller, we had similar biochemical control rates in the community hospital setting.

For our comparisons, the overall survival was greater for Cs-131 compared to Pd-103 and I-125 groups combined (p =0.25; Figure 2), but this was not statistically significant. This may be due to only 8 deaths in the Cs-131 group and shorter follow-up. It is our view that absolute differences in all-cause mortality here may be attributed to intercurrent disease. The overall survival in the Cs-131 group was similar to that reported by Moran et al. [13].

Finally, dosimetric comparison between isotopes revealed Cs-131 dosimetry qualitatively similar to I-125 and Pd-103. Prescription doses followed standard recommendations [15]. We found that for the same dose coverage of the prostate (D90), statistically greater V150 and V200 was observed using I-125 than Cs-131 (Figure 3). The delivered activity per seed and the number of seeds employed were greater compared to other isotopes. Corrected total source strength showed a similar distribution for Cs-131 compared to I-125 and Pd-103 with mean percent V150 of 36% for both I-125 and Pd-103 versus 27% for Cs-131 (p<0.0001) consistent with superior homogeneity with use of Cs-131. By comparison, Yang et al. [16] replanned cases previously implanted with I-125 using Cs-131 and Pd-103 prescriptions. They found that Cs-131 allowed for improved dose homogeneity with sparing of urethra and rectum and comparable or fewer seeds and needles required. This would suggest that Cs-131 may offer some dosimetric advantages compared with the other isotopes. It is clear that careful attention to patient selection and treatment planning will be important for clinical use of Cs-131 due to its high initial dose rate. 

Permanent interstitial brachytherapy with or without external beam radiation is an attractive option for men presenting with non-metastatic prostate cancer. This study suggests similar PSA failure rates and overall survival for Cs-131 along with dosimetric equivalence compared to traditional isotopes [17,18]. 

This study has significant limitations which are related mainly to the fact that the patients were treated over a relatively long time period. We were not able to match the three groups and the risk category for the prostate cancer in the Cs-131 group was clearly lower. In the current analysis, we did not study the correlation between GU and GI toxicities and doses delivered to urethra and rectum. Other drawbacks of the study include relatively small numbers of cases, retrospective analysis, and shorter follow-up for PSA failure among the Cs-131 cases (27 months versus 40 months for I-125 and Pd-103). Nonetheless, with this experience in the community hospital setting, we continue to offer Cs-131 prostate brachytherapy alone or combined with external radiation and/or hormone therapy as a standard option based on a patients’ risk category. 

This work has previously been presented in part in abstract form at the 2020 American Society of Clinical Oncology Genitourinary Oncology Meeting and at the 2018 Michigan Cancer Consortium Annual Meeting [17,18]. 

## Conclusions

Permanent interstitial brachytherapy is an attractive option for select men presenting with low-risk and favorable intermediate-risk prostate cancer. This study compared three groups of patients treated with three different commonly used isotopes and represents the excellent outcomes that can be obtained in a community hospital system with these methods. There was excellent PSA control for Cs-131 patients similar to or better than I-125 and Pd-103 patients at 5 years. We also report that overall survival was not statistically different between the three groups of patients. Based on the shorter half-life, employing the Cs-131 isotope may lead to a more rapid resolution of side-effects. For these reasons, we have changed our practice and offer Cs-131 based implants to most patients who are candidates for seed implants. 
